# "Letting myself go forward past wrongs": How regulatory modes affect self-forgiveness

**DOI:** 10.1371/journal.pone.0193357

**Published:** 2018-03-12

**Authors:** Antonio Pierro, Gennaro Pica, Anna Maria Giannini, E. Tory Higgins, Arie W. Kruglanski

**Affiliations:** 1 University of Rome “La Sapienza”, Rome, Italy; 2 Columbia University, New York, NY, United States of America; 3 University of Maryland, College Park, MD, United States of America; Landstinget Blekinge, SWEDEN

## Abstract

The present research addresses the question of whether regulatory-mode orientations affect self-forgiveness. We expected that people with a strong locomotion orientation would be more inclined to self-forgiveness because of their tendencies toward movement and change, which focus them on the future, whereas people with a strong assessment orientation would refrain from self-forgiveness due to their evaluative tendencies which focus them on the past. These hypotheses were supported by the results in four studies that tested the relation between regulatory modes and self-forgiveness by measuring (Studies 1, 3 and 4) and manipulating (Study 2) regulatory-mode-orientations. Finally, in Study 4 we examined more closely our hypothesis that the relation between self-forgiveness and regulatory modes is mediated by past and future temporal foci. The implications of the results for regulatory mode theory are also discussed.

## Introduction

All of us, at least once in our life, has behaved badly and felt guilty later. For instance, you may have offended someone or hurt their feelings, made mistakes that harmed others, or did something you knew was wrong at the time. All such situations have the potential to produce negative thoughts and feelings toward the self [1]. For example, people may begin to dislike themselves and believe themselves not worthy of trust and love, may reduce their self-esteem, or may even suffer affective disorders. In order to cope with these destructive consequences of wrongful actions and to move on, people may need to forgive themselves. However, not all of us forgive ourselves and turn our gaze to the future, and, if we do, we don’t do it all of the time. How do we deal with past wrongdoings and what are the antecedents of forgiving the self? The aim of the present research is to address these questions and increase our understanding of the antecedents of self-forgiveness.

Given that self-forgiveness involves some sort of motivational change that involves self-regulation, it should be useful to consider self-forgiveness from the perspective of self-regulatory mechanisms. We propose that differences in the self-regulatory orientations of locomotion versus assessment [2] may relate to differences in self-forgiveness. Specifically, locomotors’ tendency to change from a state to state and move forward leads to a greater inclination to be self-forgiving. In contrast, assessors‘ tendency to critically evaluate and compare their current state with previous experiences leads to a lesser inclination to be self-forgiving.

### Self-forgiveness: Antecedents, correlates and consequences

Self-forgiveness has been defined as a positive attitudinal shift in the feelings, actions, and beliefs about the self, following a self-perceived transgression or wrongdoing committed by the self [3–5]. Thus, forgiving the self can be considered as an adaptive mechanism of humans that helps them to restore a positive sense of the self and safeguards their overall well-being against the toxic effects of guilt, shame and regret [6–8]. A transgression from normative rules or offences toward other people with unwanted consequences may in fact elicit psychological distress that needs to be reduced. Self-forgiveness may help to achieve such a restoration by limiting self-punishment, self-condamnation, and, instead, increasing benevolence towards the self [8]. Consistent with this reasoning, research has found that self-forgiveness is associated with value reaffirmation [9] and self-acceptance [10].

Empirical evidence suggests that self-forgiveness is linked with high self-esteem, low neuroticism and low levels of anxiety and depression [11–12]. Similarly, it has been found to be positively linked with positive emotions, and with a lack of shame [13]. Numerous studies have consistently demontrated that self-forgiveness has a positive impact on overall well-being [14–18]. For instance, when people self-forgive their feelings, attitudes and beliefs toward the self become more positive and this in turn leads to lower levels of depressive affect [19]. Furthermore, self-forgiveness has been found to reduce procrastination [20]. More specifically, among students who reported high levels of self-forgiveness for procrastinating studying for the first examination, procrastination on preparing for the subsequent examination was reduced. The above finding suggests that self-forgiveness for past wrongs allows for forward movement toward goal pursuit by reducing procrastination tendencies.

Because of the correlational nature of the findings in many of the past studies, it is not fully clear what the motivational antecedents of self-forgiveness might be. It seems, however, that the purpose of self-forgiveness is to protect one’s well-being (self-image and self-concept) in order to move forward optimistically, avoiding being stopped by undermining feelings of guilt, blame and regret. This view of self-forgiveness is consistent with McCullough and collegues‘[21–22] definition of forgiveness as a suite of *motivational changes* that occurs following a transgression whereby the victim becomes less motivated by revenge and more motivated toward benevolence. Even though this definition refers to interpersonal forgiveiness, it is fairly likely to assume that the same *motivational changes* occur in the process of self-forgiveness whereby the person shifts from feeling guilty to benevolence toward the self. If this is the case, then self-forgiveness would be supported by a motivational orientation that encourages change, thus allowing movement towards the future. As we will see next, this describes the motivational orientation of the locomotion mode.

### Locomotion and assessment orientations

Regulatory mode theory [2, 23–24]captures two critical dimensions of goal-directed behavior, *assessment* and *locomotion*. *T*he *assessment* dimension refers to “the comparative aspect of self-regulation concerned with critically evaluating entities or states, such as goals or means, in relation to alternatives in order to judge relative quality” [2] (p.794). The *locomotion* dimension refers to “the aspect of self-regulation concerned with movement from state to state and with committing the psychological resources that will initiate and keep goal-related movement in a straightforward and direct manner, without undue distractions and delays” (p. 794). In other words, the *assessment* function is to select the right or best course of action in goal pursuit, whereas the *locomotion* function is to move on, effect change, and manage action towards the desired end-state.

Locomotion and assessment modes can operate as individual difference variables, but they can also be situationally induced [25]. Thus, although assessment and locomotion can work together as parts of the same goal pursuit orientation [26], they are also conceived as essentially orthogonal dimensions, each of which can receive differential emphasis to same phenomena [23]. Empirical evidence shows that assessment positively correlates with fear of invalidity, discomfort with ambiguity, neuroticism, low self-esteem, and negative mood [2]. Locomotion, on the other hand, positively correlates with psychological vitality, self-esteem, optimism, and being decisive, and it negatively correlates with social anxiety and depression [2].

Furthermore, assessors, compared to locomotors, are more inclined to experience nostalgia [27] and suffer more from counterfactual thinking after failure and from experiencing regret about their choices [28]. More specifically, Pierro and colleagues [28] for example, found in a series of studies that high (*vs*. low) assessors were more likely to engage in counterfactual thinking and this produced more regret about a previous negative decision (e.g., purchasing a faulty product with poor customer service). In contrast, high locomotors were less likely to engage in counterfactual thinking or experience regret from a past mistake. Whereas assessors’ concern with making the right choice through critical evaluation orients them toward past experiences, considering and reconsidering the consequences of past actions to understand the right way to proceed [27–28], locomotors’ concern with effecting change and moving focuses them on present and future possibilities [2, 24, 29]. The above regulatory concerns can have secondary consequences: assessment may leave people confined in the current state, evaluating the past and comparing it with the present, potentially creating repercussions for self-forgiveness; whereas locomotion may help to overcome the past mistakes and effectively move forward, potentially more easily leading to self-forgiveness. Consistent with this difference, assessment has been found to be positively related to procrastination whereas locomotion has been found to be negatively related [30]. Notably, procrastination is negatively linked to self-forgiveness [20]. All of this suggests that there could be a positive link between locomotion and self-forgiveness and a negative link between assessment and self-forgiveness.

### The present research

The purpose of the present research was to test whether individuals’ self-regulatory mode orientation affects self-forgiveness. The theory and the findings described above suggest that people with a strong locomotion orientation are inclined to shift their attention away from what happened in the past, effect change and move forward to attain goals in the future. We hypothesize that these locomotion tendencies toward change and movement promotes self-forgiveness. In contrast, people with a strong assessment orientation are inclined to evaluate their past experience in order to make the right choice in the present. A consequence of this is that they may keep in mind the past and thus enhance their tendencies toward stasis over dynamic action and change. Thus, we hypothesize these assessment concerns would reduce self-forgiveness. These predictions should hold true whether individuals’ heightened locomotion or assessment states derive from chronic predispositions to be in these states or from situational forces that momentarily induce them.

To test these hypotheses, we conducted a series of four studies whereby we tested the general association between regulatory modes and self-forgiveness, using different methods and measures. Specifically, we measured (in Studies 1, 3 and 4) and manipulated (in Study 2) regulatory mode and then we measured self-forgiveness. Finally, in Study 4 we examined more closely our hypothesis that the relation between self-forgiveness and the regulatory modes is mediated by the different attention locomotors and assessors give to thinking about the future or the past. This set of studies was approved by the Ethical Committee of the department of developmental and socialization processes (University of Rome “La Sapienza”) under protocol 63-11/23, titled: “The influence of Regulatory Mode Orientations on Self-Forgiveness”.

## Study 1

Our first study examined the basic relationships between chronic locomotion and assessment orientations and self-forgiveness. We hypothesized that people with a strong locomotion orientation would be more inclined to self-forgive their past misdeeds; while people with a strong assessment orientation would be more resistant to self-forgive their own wrongs.

## Method

### Participants

On a volunteer basis, a total of 323 Italian people (192 women) participated in the study. Their *M*_*age*_ was 30.06 (*SD*_*age*_ = 10.93). The total sample consisted of two separate sub-samples. Sample 1 consisted of 168 university students (103 women). Their *M*_*age*_ was 23.73 (*SD*_*age*_ = 2.89). Sample 2 consisted of 155 non-students (89 women), with *M*_*age*_ of 36.92 (*SD*_*age*_ = 12.24). This second non-student sample comprised 81 (52.3%) white-collar employees, 52 (33.5%) professionals, 4 (2.6%) entrepreneurs, 10 (6.5%) unemployed, and 8 (5.2%) pensioners. Other than the differences on student and professional status, the two sub-samples also significantly differ on age, non-students sample being older than the students sample, *t*(321) = -13.56, *p* < .001. As previous research showed that older adults are more forgiving than younger adults [31], we decided to run the analyses separately on each sub-sample as well as on the total sample, controlling for gender and age.

### Procedure

All participants filled out the Locomotion and Assessment scales. They then completed a measure designed to assess self-forgiveness.

## Measures

### Locomotion and assessment orientations

The Italian versions of the Locomotion and Assessment Scales [2] constitute of two separate 12-item self-report measures designed to tap individual differences in these tendencies. Specifically, respondents rated the extent to which they agree with self-descriptive statements reflecting locomotion (e.g., "By the time I accomplish a task, I already have the next one in mind") and assessment (e.g., I spend a great deal of time taking inventory of my positive and negative characteristics"). Ratings were made on a 6-point Likert type scale with the response alternatives anchored at the ends with 1 (*Strongly Disagree*) to 6 (*Strongly Agree*). We computed two composite scores (one for Locomotion and one for Assessment) by averaging across responses to each item. For the total sample the Cronbach ‘s α for the locomotion scale was .78 and the α for the assessment scale was .75. In this total sample, the two scales were not correlated (*r* = -.08; n.s.), consistent with previous studies [2]

### Assessing self-forgiveness

All participants responded to 4 items derived from the feeling subscale of Wohl and colleagues’ [19] State Self-Forgiveness Scale (SFS). This state scale was created to target self-forgiveness for specific transgressions against the self as opposed to more general, or global forgiveness of the self. Specifically, following the Wohl and colleagues’ [19] procedure, participants were told to think of the most significant experience in which they did something they believe to have been wrong. To the point, participants were asked to think about an incident in which they took responsibility for committing a wrongful behavior. Instructions also specified that the event should involve an incident that was hurtful to someone else. Participants were told to take a moment to consider the circumstances of that event and recall as much detail about what they did that was wrong. They were asked not to write what the incident entailed but instead to think about the incident and the feelings that the incident arouses in them and then complete the scale while thinking how they feel about themselves “right now” regarding the wrongful event. The 4-item scale asked for the respondents’ current feelings about their wrongdoing: I feel… rejecting of myself (Item Reversed, R), accepting of myself, dislike toward myself (R), forgiving myself. Ratings were made on a 4-point scale with the response alternatives anchored at the ends with 1 (*Not at all*) to 4 (*Completely*). The 4 items were averaged to create a composite score (Total sample Cronbach’s *α* = .72). Higher scores reflected greater self-forgiveness.

## Results

Descriptive statistics and correlations among variables for total sample and for each sub-sample are reported in Table 1. Note that in each sub-sample and in the total sample the self-forgiveness measure was positively and significantly related to locomotion, but negatively and significantly related to assessment. Also note that the relationship between locomotion and self-forgiveness as well as between assessment and self-forgiveness in the two sub-samples did not significantly differ (*Z* = 1.042, n.s.; and *Z* = .382, n.s., respectively).

**Table 1:** Descriptive and correlations between variables (Study 1).

	M	SD	1	2	3
Sample 1 (Students, N = 168)					
Locomotion	4.50	.70	(.81)		
Assessment	3.74	.79	-.20**	(.76)	
Self-Forgiveness	2.94	.68	.30***	-.28***	(.73)
Sample 2 (Non-Students, N = 155)					
Locomotion	4.76	.64	(.73)		
Assessment	3.65	.78	.08	(.74)	
Self-Forgiveness	3.13	.62	.19*	-.24**	(.70)
Total Sample (N = 323)					
Locomotion	4.62	.68	(.78)		
Assessment	3.70	.79	-.08	(.75)	
Self-Forgiveness	3.03	.66	.27***	-.27***	(.72)

*p < .05

**p < .01

***p < .001.

In bracket (Cronbach’s *α*).

Predictions regarding the differential and unique effects of locomotion and assessment orientations on self-forgiveness were tested by means of three separate multiple regression analyses: one for each sub-sample and one for the total sample. In these analyses we regressed the self-forgiveness scores on both the locomotion and assessment indices as predictors. Gender (dummy coded; Male = 0; Female = 1) and age were entered as control variables. For the total sample we also entered sample (dummy coded; Student = 0; non-Student = 1) as control variable. Summary of results of these analyses are reported in Table 2.

**Table 2:** Summary of multiple regression analyses (Study 1).

	Sample 1 (Students)	Sample 2 (Non-Students)	Total Sample
	*β*	*β*	*β*
Locomotion	.25***	.21**	.23***
Assessment	-.23**	-.26***	-.25***
Gender	.02	-.01	.01
Age	.03	-.02	-.01
Sample	-	-	.09

**p < .01

***p < .001.

Gender (Male = 0; Female = 1); Sample (Students = 0; Non-students = 1).

As Table 2 shows, for each sub-sample, as well as for total sample, the pattern of results was the same. Specifically, as expected, self-forgiveness was significantly and positively related to locomotion (sample 1, *β* = .25, *p* < .001; sample 2, *β* = .21, *p* < .01; Total sample, *β* = .23, *p* < .001, respectively) and significantly and negatively related to assessment (sample1, *β* = -.23, *p* < .001; sample 2, *β* = -.26, *p* < .001; Total sample, *β* = -.25, *p* < .001, respectively).

## Study 2

The purpose of the second study was to replicate the results of Study 1, this time using an experimental manipulation of locomotion and assessment. This replication is important because regulatory mode theory assumes the functional equivalence of individual difference measures and situational inductions of the locomotion and assessment orientations. Additionally, the manipulation of regulatory mode allows for a stronger causal inference to be drawn between assessment/locomotion and subsequent experience of self-forgiveness.

## Method

### Participants

97 university students (55 women) in Italy participated in the study on a volunteer basis. Their *M*_*age*_ *was* 23.07 (*SD*_*age*_ = 2.91).

### Procedure and materials

Participants were randomly assigned to the locomotion, assessment, and control conditions. Following Avnet and Higgins’ [25] procedure, locomotion and assessment were manipulated by asking participants to complete a ‘‘personal memories” questionnaire by which participants will be asked to think of three different situations in which they personally exemplified either high locomotion or high assessment behaviors and to write them down. Specifically, to manipulate locomotion, participants were asked to: ‘‘Think of a day when you made many different things”; ‘‘Think of a time when you finished one project and did not wait long before you started a new one”; ‘‘Think of a time when you decided to do something and you could not wait to get started.” For assessment, they will be asked to: ‘‘Think of some occasion in which you compared yourself with other people”; ‘‘Think of some occasion in which you thought about your positive and negative characteristics”; ‘‘Think of some occasion in which you critiqued work done by others or yourself.” These situations were taken from items in the locomotion and assessment scales of the regulatory mode questionnaire [2]. Avnet and Higgins [25] have shown that this experimental manipulation of locomotion and assessment states is effective in inducing the corresponding psychological states.

After completing the “personal memories” questionnaire, which experimentally induced the locomotion and assessment tendencies, participants completed the same 4-item scale of self-forgiveness used in study 1. Control group participants did not complete the personal memories questionnaire and instead were immediately asked to complete the self-forgiveness scale. In this study the self-forgiveness Cronbach ‘s *α* was .72 (*M* = 2.84, *SD* = .66).

## Results

As predicted, a one-way ANOVA, with age and gender (dummy coded; Men = 0; Women = 1) as covariates, yielded a significant effect of experimental conditions on self-forgiveness, *F*(2, 92) = 7.90, *p* = .001, *pη*^*2*^ = .15, while age and gender did not affect self-forgiveness [*F* (1,92) = .54, *p* = .46, *pη*^*2*^ = .01; *F* (1,92) = .75, *p* = .39, *pη*^*2*^ = .01, respectively]. Simple contrasts revealed that participants in the locomotion condition showed greater self-forgiveness (*N* = 33, *M* = 3.15, *SD* = .48) than participants in either the assessment orientation condition (*N* = 33, *M* = 2.55, *SD* = .57), *t* (94) = 3.95, *p* = .00, *Cohen’s d* = 1.14, or the control group condition (*N* = 31, *M* = 2.83, *SD* = .77), *t* (94) = 2.08, *p* = .04, *Cohen’s d* = .50. Furthermore, participants in the assessment orientation condition (though only approaching significance) showed lower self-forgiveness than participants in the control group condition, *t* (94) = 1.80, *p* = .07, *Cohen’s d* = .41.

## Study 3

In Study 1, we found that assessment was negatively related and locomotion positively related to self-forgiveness. In Study 2, we induced locomotion or assessment regulatory mode and we found that the individuals under condition of high locomotion were more likely to experience self-forgiveness than those under high assessment. A possible limit of the first two studies is that the effects of regulatory mode orientations on self-forgiveness were not controlled for possible effects of secondary variables possibly having a role in the process. For instance, we reasoned that locomotion influences self-forgiveness because of its tendency to effect change and move forward. However, one may suspect that locomotors, in order to go head and quickly move on, may recall less severe transgressions or accept less responsibility with respect of the fact, thus enhancing the ease of the self-forgiveness process. In other words, it might be possible that people with strong locomotion concerns forgive themselves more also because they do not fully admit their responsibility for the wrongful episode. On the other hand, we reasoned that assessment should block self-forgiveness because of its evaluative tendencies that lead people to remain anchored to past errors. Again, this logic may lead one to expect that assessors, being focused on past mistakes by their comparative and evaluative tendencies, may recall more severe transgressions and accept more responsibility for the wrongs recalled thus reducing the ease of the self-forgiveness process. Furthermore, as it is possible that forgive the self with respect to errors of the remote past may be easier (because these errors may lose their power and may be already overcome), we controlled for the time passed from the recalled episode and for positive and negative affect that may be induced by the recalled transgressions which both may influence self-forgiveness.

To rule out the above possibilities, in the present study we asked participants to assess their assumption of responsibility and the severity of the recalled transgressions, and we assessed the objective severity of the participant’s recalled transgressions by having two independent raters evaluating the written descriptions of the event. We expected that the relation between regulatory mode orientations and self-forgiveness should be maintained regardless of participants’ acceptance of responsibility, the severity of the transgressions recalled, the time passed since the episode occurred and regardless participants’ affect elicited by the recalled transgression. The purpose of Study 3 was to confirm the relationship between regulatory modes and self-forgiveness using a different recall task and by controlling for these possible variables.

## Method

### Participants

Eighty-five students (60 women; *M*_*age*_ *=* 23.16, *SD*_*age*_ = 3.92) participated in the experiment. The same measure of self-forgiveness used in previous two studies was the dependent variable.

### Procedure

The study proceeded in two phases. During the first phase, participants completed in class the same locomotion and assessment scales used in Study 1. In the second phase, approximately 1 month later, participants were asked to think back to an episode where they had offended or hurt someone and to briefly describe it. The instructions were as follows: “Every now and then, most or all people have hurt somebody else. We ask you to think about an episode where you offended or hurt someone”. After receiving these instructions, participants were asked to write a paragraph about the offense. The writing part served to induce participants to bring to mind the episode and their feelings about it. The written descriptions of offenses reflected a wide variety of interpersonal situations (e.g., hurt the partner, a family member, a friend). After describing the offense, participants were carefully instructed to answer the PANAS scale, the same 4 items assessing state self-forgiveness used in Study 1, the transgression severity, the time passed since the episode occurred and the acceptance of responsibility for the wrong recalled. At the end of this task, they were debriefed, thanked and dismissed.

## Measures

### Locomotion and assessment orientations

Participants’ locomotion and assessment orientations were measured with the same Italian version of the regulatory mode scale [21] used in Study 1. The Cronbach ‘s *α* for the locomotion scale was .83 and the α for the assessment scale was .71. Consistent with Study 1 and previous studies [2], the two scales were not correlated (*r* = .12; *p* = .25).

### Panas

The Panas (Positive and Negative Affect Schedule) [32] was used to gauge positive (e.g., active, proud, determined) and negative (e.g., upset, guilty, distressed) emotional responses to the transgression reported. Specifically, participants used a Likert-type scale ranging from 1 (*not at all*) to 7 (*extremely*) to endorse each of 20 adjectives in response to the question: “How do you feel currently about this transgression?”. These were then separately collapsed into the positive (*M* = 2.61, *SD* = .72, *α* = .83) and negative (*M* = 2.42, *SD* = .82, *α* = .90) affectivity subscales used in our analysis.

### Assessing self-forgiveness

All participants responded to the same 4 items used in Study 1 (*M* = 3.14, *SD* = .59, *α* = .74).

### Assessing subjective and objective trasgression severity

Participants were asked to rate using a ten point scale (1 = *not at all*; 10 = *completely*) the extent to which they saw the described offense as: (a) serious and (b) harmful or damaging to the other person [7]. The two items were correlated, *r* = .34, *p* = .002, and were averaged to assess *subjective* transgression severity. In order to also assess the *objective* transgression severity, two independent raters, naïve to the study’s hypotheses, were asked to rate the written offenses using the same two items described above. Within-rater internal consistency (Pearson’s *r*s) was .63 for rater 1 and .71 for rater 2, and averaged .73. The inter-rater agreement (Pearson’s *r*s) for the two scales was .53 for “serious”, and .52 for “harmful”, and averaged .59.

Furthermore, the correlation between the subjective and objective indexes of transgression severity were highly correlated (*r* = .54; p < .001). In addition, we used the rwg*(j)* as an index of inter-rater (subjective score, and objective raters 1 and 2 scores) agreement for multiple items [33]. The value obtained of .93, calculated using a normal distribution, suggested high inter-rater agreement. Thus, we calculated a total index of transgression severity by adding the subjective and objective transgression severity scores.

### Responsibility and the time passed since the incident occurred

Five items [11], rated from 0 (*completely disagree*) to 10 (*completely agree*), assessed the degree to which participants felt responsible for the offense (*α* = .82). Statements included: ‘‘I feel I was responsible for what happened,” ‘‘I wasn’t really to blame for this” (Reverse scored), ‘‘I was in the wrong in the situation,” ‘‘This was clearly my fault,” and ‘‘I did not really do anything wrong” (R). They also reported how long ago the incident occurred (i.e., in months; *M* = 24.14; *SD* = 34.61).

## Results

Descriptive statistics and correlations among variables are reported in Table 3. Note that the self-forgiveness measure was positively and significantly related to locomotion, but negatively and significantly related to assessment. Furthermore, transgression severity, time passed since the transgression occurred and acceptance of responsibility of the wrong were not related to regulatory mode orientations, helping to rule out the possibility that high-locomotors simply recall less severe transgressions and do not fully accept their responsibility for the past wrong; and that high-assessors simply recall more severe transgressions and attribute to the self a greater responsibility for the offense reported.

**Table 3:** Descriptive and correlations between variables (Study 3).

		M	SD	1	2	3	4	5	6	7	8	9	10
(N = 85)													
Self-Forgiveness		3.14	.59	(.74)									
Locomotion		4.36	.64	.28**	(.83)								
Assessment		3.76	.63	-.24*	.12	(.71)							
Transgression Severity ^		7.15	0.97	-.08	.03	-.10	(.93^+^)						
Acceptance of Responsibility		7.45	1.63	-.16	-.05	.07	.34**	(.82)					
Positive affect		2.61	.72	.31**	.09	.04	.07	.05	(.83)				
Negative affect		2.42	.82	-.39***	-.19	.05	.26*	.07	-.03	(.90)			
Time		24.14	34.62	.07	.16	.05	.20	.06	-.23*	-.15	-		
Age		23.16	3.92	.23*	.13	-.11	.03	-.01	.07	-.22*	.52***	-	
Gender		-	-	.07	.10	.02	-.13	.02	.04	-.03	.01	-.07	-

***p < .001

**p < .01

*p < .05.

In bracket (Cronbach’s *α*)

^+^rwg(j) index.

^ Total (subjective and objective) transgression severity index.

Predictions regarding the differential and unique effects of locomotion and assessment orientations on self-forgiveness were tested by means of a multiple regression analyses. In this analysis we regressed the self-forgiveness scores on both the locomotion and assessment indices as predictors. Gender (dummy coded; Men = 0; Women = 1), age, transgression severity, assumption of responsibility, time occurred since the transgression, positive and negative mood were entered as control variables. Nor the effects of transgression severity (*β* = -.02, *t* = -.19, *p* = .85), the time passed since the episode occurred (*β* = .06, *t* = .47, *p* = .64) and the acceptance of responsibility (*β* = -.12, *t* = -1.24, *p* = .22), neither the effects of age (*β* = .07, *t* = .59, *p* = .56) or gender (*β* = .03, *t* = .34, *p* = .74) were significant. As expected, self-forgiveness was positively related to positive affect (*β* = .31, *t* = 3.06, *p* = .003) and negatively related to negative affect (*β* = -.30, *t* = -2.93, *p* = .004). More importantly, the relation between self-forgiveness and the two regulatory mode orientations remain significant after controlling for all the above variables: self-forgiveness was positively related to locomotion (*β* = .20, *t* = 2.01, *p* = .048) and negatively related to assessment (*β* = -.25, *t* = -2.59, *p* = .01). We also run two separate regression analyses as described in the text, one controlling for *subjective* and one controlling for *objective* indexes of transgression severity. The pattern of results was the same for both the objective and subjective indexes as well as for the total severity transgression index.

## Study 4

The previous three studies, using different methods, offer consistent evidence that locomotion and assessment are associated with self-forgiveness in opposite directions: locomotion is positively associated with self-forgiveness whereas assessment is negatively associated with self-forgiveness. Notably, our proposal was that these two relations are mediated by different forces; specifically a difference in temporal focus. The purpose of Study 4 was to investigate this difference. We hypothesized that the general association between regulatory modes and self-forgiveness is mediated by the attention locomotors and assessors give to thinking about the future or the past: Locomotion should be positively associated with self-forgiveness because of the locomotors’ tendency to focus on the future, whereas assessment should be negatively associated with self-forgiveness because of assessors’ tendency to focus on the past.

In summary, the purpose of the fourth study was to replicate the results of Study 1 and Study 3, this time using a dispositional self-forgiveness scale, and, more important, to verify the mediating role of temporal focus on the relations between regulatory modes and self-forgiveness.

## Method

### Participants

On a volunteer basis, a total of 189 students from University of Rome “La Sapienza” (133 women) participated in the study. Their *M*_*age*_ was 24.53 (*SD*_*age*_ = 2.81).

### Procedure

All participants filled out the Locomotion and Assessment scales followed by temporal focus scale. They then completed a measure designed to assess dispositional self-forgiveness tendency.

## Measures

### Locomotion and assessment orientations

Participants responded to the same Italian versions of the Locomotion and Assessment Scales used in previous studies. For the present sample the Cronbach ‘s *α* for the locomotion scale was .83, and the α for the assessment scale was .77. *M* of the locomotion score was 4.37 (*SD* = .62) and *M* of the assessment score was 3.65 (*SD* = .67). Again, in this sample, the two scales were not correlated (*r* = -.05; *p* = .53), consistent with previous studies [2].

### Assessing temporal focus

Participants completed the 12-item Temporal Focus Scale (TFS) (four items each for the past, present, and future), which was developed by Shipp, Edwards and Lambert [34]. Illustrative items include ‘‘I replay memories of the past in my mind” for past temporal focus; ‘‘My mind is on the here and now” for present temporal focus; and ‘‘I focus on my future” for future temporal focus. The TFS items were rated on a 7-point scale describing the frequency with which the respondent thought about the time frame indicated by the item (1 = *Never*; 7 = *Constantly*). We computed three composite scores (one for each temporal focus subscale) by averaging across responses to each item. The Cronbach ‘s *α*for the past temporal focus scale was .91, the *α*for the present temporal focus scale was .85, and the *α* for the future temporal focus scale was .92.

### Assessing self-forgiveness

All participants responded to the 6 items derived from the dispositional Self-Forgiveness subscale of the Heartland Forgiveness Scale (HFS) developed by Thompson et al. [35]. Specifically, following the Thompson et al. [35] procedure, participants were told that “in the course of our lives negative things may occur because of our own actions. For some time after these events, we may have negative thoughts or feelings about ourselves.” To the point, participants were asked to think about how they *typically* respond to such negative events and, then, to complete the following 6 items: “Although I feel bad at first when I mess up, over time I can give myself some slack”; “I hold grudges against myself for negative things I’ve done” (R); “Learning from bad things that I’ve done helps me get over them”; “It is really hard for me to accept myself once I’ve messed up” (R); “With time I am understanding of myself for mistakes I’ve made”; “I don’t stop criticizing myself for negative things I’ve felt, thought, said, or done” (R).

Ratings were made on a 7-point scale with the response alternatives anchored at the ends with 1 (*Almost Always False of Me*) to 7 (*Almost Always True of Me*). The 6 items were averaged to create a composite score (*α* = .70; *M* = 4.43, *SD* = .94). Higher scores reflect greater dispositional self-forgiveness tendency.

## Results

Descriptive statistics and correlations among variables are reported in Table 4.

**Table 4:** Descriptive and correlations between variables (Study 4).

	M	SD	1	2	3	4	5	6
(N = 189)								
Locomotion	4.37	.62	(.83)					
Assessment	3.65	.67	-.05	(.77)				
Past temporal focus	5.10	1.09	-.12^+^	.41***	(.91)			
Present temporal focus	5.12	1.00	.25***	.003	-.09	(.85)		
Future temporal focus	5.54	1.05	.37***	.02	.004	.33***	(.92)	
Self-Forgiveness	4.43	.94	.22**	-.27***	-.40***	.19**	.25***	(.70)

**p < .01

***p < .001

^+^ *p* = .09.

In bracket (Cronbach’s *α*).

As in previous studies, predictions regarding the differential and unique effects of locomotion and assessment orientations on self-forgiveness tendency were tested by means of a multiple regression analysis. In this analysis we regressed the self-forgiveness scores on both the locomotion and assessment indices as predictors. Gender (dummy coded; Men = 0; Women = 1) and age were entered as control variables.

As expected, self-forgiveness, after controlling for gender (*β* = -.25, *p* < .001) and age (*β* = -.07, *p* = .30), was significantly and positively related to locomotion (*β* = 23, *p* < .001) and significantly and negatively related to assessment (*β* = -.28, *p* < .001).

### A mediational analysis: Multiple mediator model

As anticipated, we also tested the hypothesized mediating role of temporal focus in the relationship between regulatory modes and self-forgiveness. We expected the positive relation between locomotion and self-forgiveness to be mediated by the future temporal focus, and the negative relation between assessment and self-forgiveness, by the past temporal focus.

**Fig 1:**
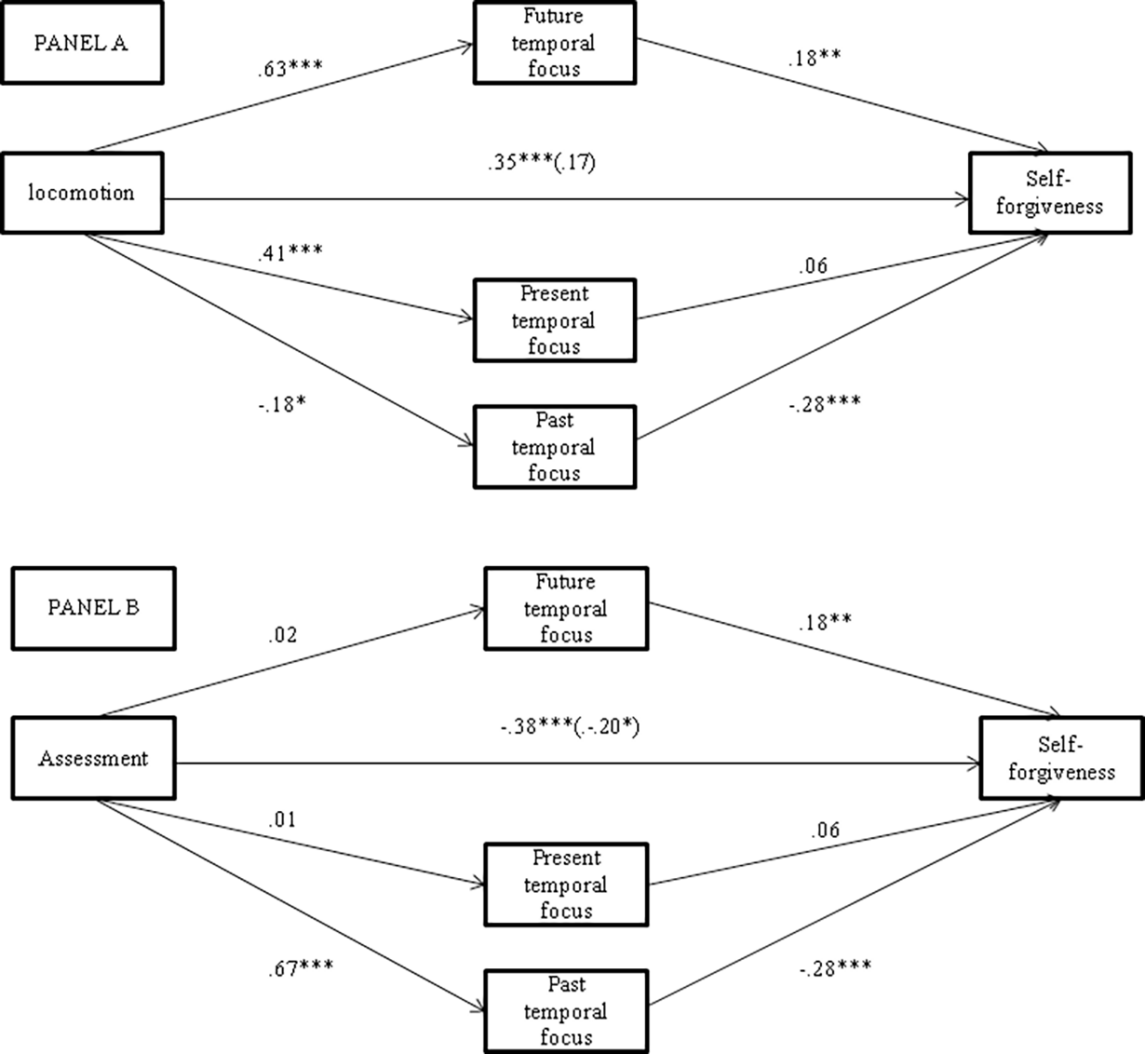
**Coefficients (*b* values) representing effects of locomotion (Panel A) and assessment (Panel B) orientations on mediators and self-forgiveness (Study 4).** Note: The effects of Gender and Age were omitted for a better clarity of the figure. The value in parentheses represents the direct effect of regulatory modes on self-forgiveness after controlling for mediating variables. **p < .01; ***p < .001; ^+^*p* = .13.

To test these mediational hypotheses we estimated a model where the potential mediators (past, present and future temporal focus in our case) controlled for one another and that includes multiple independent variables (locomotion and assessment modes in our case). To examine the proposed mediation model, we employed Preacher and Hayes' [36–37] procedure to extrapolate estimates of direct and indirect effects. Preacher and Hayes’ strategy employs the use of bootstrapping, a non-parametric re-sampling procedure, to estimate the size of indirect effects. The present analysis was performed using PROCESS program (Model 4) [38]. As specified by Hayes, PROCESS (Model 4) can be used to estimate the coefficients in a multiple mediation model with multiple independent variables, although it provides no information that can be used to test a combined indirect effect involving all independent variables. Nevertheless, covariates are mathematically treated exactly like independent variables in the estimation, with paths to all mediators and the outcome, so if the desired model has *k* independent variables, PROCESS can be run *k* times, each time listing one variable as the independent variable and treating remaining *k–* 1 independent variables as covariates. Each run of PROCESS generates the effects (including indirect effects) for the variable currently listed as independent variable. Then, following this procedure to extrapolate estimates of direct and indirect effects, we performed two multiple mediation analyses testing a model with the mediating role of past, present and future temporal focus: one for the effect of locomotion on self-forgiveness and one for the effect of assessment on self-forgiveness. For each of the regulatory mode effects, we controlled for the alternative mode’s effect, included in the model as a covariate. Also gender and age were included in the models as control variables. Ninety-five percent confidence intervals (CI) were employed and 1000 bootstrapping re-samples were run. Confidence intervals were adjusted for bias (bias corrected, BC). The results obtained from the two analyses are summarized in Table 5 and in Fig 1.

**Table 5:** Indirect effects of regulatory mode orientations on self-forgiveness through proposed mediators (Study 4).

			Bias-corrected bootstrap CIs	
Mediator	Bootstrap effect	*SE*	Lower	Upper
Mediated effect of locomotion				
Total indirect effect	.19.05.03.11	.06	.09	.32
Past temporal focus		.04	-.01	.13
Present temporal focus		.03	-.02	.08
Future temporal focus		.04	.04	.21
Mediated effect of assessment				
Total indirect effect	-.18-.19.00.00	.05	-.30	-.09
Past temporal focus		.05	-.31	-.10
Present temporal focus		.01	-.02	.03
Future temporal focus		.02	-.03	.04

*Note*. CI = Confidence Interval.

As Fig 1 shows, of the three potential mediators, the future temporal focus was significantly and positively related to self-forgiveness and the past temporal focus was significantly and negatively related to self-forgiveness. No significant effect was found for present temporal focus.

The results obtained from the analysis regarding locomotion mediated effect shows that the *total* effect of locomotion on self-forgiveness was significant and positive, *b* = .35, *SE* = .10, *p* < .001. Moreover, as Fig 1 (Panel A) shows, the *direct* effect of locomotion become *non-significant* (*b* = .17, *SE* = .10, *p* = .11) after controlling for the mediators. Concerning the relationship between locomotion and the three potential mediators, locomotion was significantly and positively associated with future and present temporal focus and negatively (although only marginally significant) with past temporal focus. Finally, as Table 5 shows, the *total indirect* effect of locomotion on self-forgiveness was significant. More importantly, of the three *indirect* effects, only the effect of locomotion on self-forgiveness through the future temporal focus was significant (indirect effect = .11, with BC 95% CI of .0368 to .2112, not containing zero).

The results obtained from the analysis regarding assessment mediated effect shows that the *total* effect of assessment on self-forgiveness was significant and negative (*b* = -.38, *SE* = .09, *p* < .001). Moreover, as Fig 1 (Panel B) shows, the *direct* effect of assessment remain significant (*b* = -.20, *SE =* .10, *p =* .04) although significantly *reducted* when controlling for the mediators. Assessment was significantly and positively associated only with the mediating variable of past temporal focus. Finally, and more importantly, of the three *indirect* effects, only the effect of assessment on self-forgiveness through past temporal focus was significant (indirect effect = -.19, with BC 95% CI of -.3086 to -.1046, not containing zero). Overall, the multiple mediator model was highly significant, *F* (7,181) = 11.47, *p* < .001, with *R*^2^ = .31. These results are highly consistent with our mediational hypotheses.

## General discussion

The major purpose of the present research was to contribute to the understanding of the self-regulatory antecedents of self-forgiveness. Based on recent findings linking regulatory mode with the construct of time [29, 39], where the concept of self-forgiveness can be included as it refers to dealing with past memories (i.e., time as a *flow* from past, present and future), it was predicted that locomotion and assessment would impact self-forgiveness differently. We reasoned that intrinsic locomotion tendencies towards change and movement helps to *move forward* into the future effectively, overcoming the past and enhancing the possibilities of self-forgiveness, while assessment tendencies to appraisals and comparisons leads to a stasis state where the past is evaluated in the present, thus reducing the easy of self-forgiveness.

The results of four studies provided strong support for our hypotheses. In Study 1 we measured locomotion, assessment [2] and state self-forgiveness by means of a self-report scale, and we found a positive association between locomotion and self-forgiveness and a negative association between assessment and self-forgiveness. In Study 2 regulatory modes were manipulated and, consistent with the hypotheses, it was found that participants in the locomotion condition exhibited higher tendency towards self-forgiveness compared to participants in both assessment and control conditions; participants in the assessment condition exhibited lower tendency towards self-forgiveness compared to participants in the control condition. Study 3 confirmed the results of Study 1 helping us to rule out the possibility that, in order to move on and easily change the current state, locomotors simply referred to less severe transgressions and present less acceptance of responsibility than assessors, thus resulting in an *ease* of self-forgiveness. In fact, since self-forgiveness involves the process of admitting one’s responsibility of the wrong, controlling for the above variables we can exclude that we are not referring to what has been called pseudo-self-forgiveness, that is when people do not accept their responsibility for the error [40]. It was shown that the hypothesized relations between regulatory mode orientations and self-forgiveness remained even controlling for transgression severity, acceptance of responsibility, time passed since the recalled transgressions occurred and positive and negative mood.

Furthermore, with the aim of examine the mediating role of temporal focus in the relation between self-forgiveness and regulatory modes, the results of Study 4 also showed that the positive relation between locomotion and dispositional self-forgiveness was mediated by the future temporal focus, and the negative relation between assessment and self-forgiveness was mediated by the past temporal focus. It should be noted, however, that the mediation analysis was conducted using cross-sectional data, thus giving problems to inferring the causality between the variables [36]. However, both theoretical and methodological issues make us confident that the findings of Study 4 are consistent with (or do not contradict) our mediation hypothesis.

Theoretically speaking, consistent with the suggestion that theory might help to determine the proper order of the variables in a proposed mediation model [37, 41], our mediation hypothesis was built upon previous work showing that locomotion and assessment influence how individuals differently focus on time [29]. In particular, due to their predominant motivation towards change and movement which focuses them towards the future, high-locomotors have been found to be less nostalgic, while due to their predominant motivation towards appraisal which focuses them on assessing and comparing the past with the present high-assessors have been found to be more nostalgic [27]. In the same vein, high assessors have been found to experience more regret and counterfactual thinking, whereas high locomotors have been found to experience less counterfactuals and less regret about past decisions [28]. Furthermore, high-locomotors procrastinate less and focus on goal pursuit, while high-assessors procrastinate more [30]. Importantly, procrastination and self-forgiveness has been shown to be negatively related [20], suggesting that people who forgive themselves reduce their procrastination tendencies by focusing on immediate goal pursuing, thus allowing for movement.

Methodologically speaking, three indicators play a supportive role of our hypotheses. First, we know from Study 2 that regulatory mode orientations (being manipulated) influence self-forgiveness. Second, in Study 3 we measured the locomotion and assessment scales approximately 1 month before participants were asked to think back to answer the self-forgiveness questionnaire. Third, in Study 1 we first measured regulatory mode orientations and then self-forgiveness; and in Study 4 again we first measure regulatory mode orientations and then we measured temporal focus and self-forgiveness (in this exact temporal order).

Notwithstanding, as suggested in Preacher and Hayes [37], in order to corroborate the validity of our mediation hypothesis, and to rule out possible alternative causal models (e.g., temporal foci influence regulatory mode orientations which in turn influence self-forgiveness), might be adopted two different strategies (1) implement longitudinal designs, also including covariates (e.g., self-acceptance) to help eliminate source of spurious correlation between temporal foci (M) and self-forgiveness (Y); and (2) use the experimental-causal-chain strategy [42] to experimentally test the hypothesized mediation model by (a) manipulating regulatory mode and then measuring temporal foci, and by (b) manipulating temporal foci and then measuring self-forgiveness. Future studies may implement the above designs to corroborate the validity of the proposed mediation hypothesis. Taken together, the four studies are consistent with the proposition that locomotion inclinations lead to effectively overcoming past mistakes and move on, focusing on future goals, thus enhancing the inclination to forgive the self, while assessment inclinations lead to dig deeper to understand better what happened in past wrongs and compare that situations with the present, producing a state of stasis that obstacles the self-forgiveness process.

Although the above results are quite clear, other variables, not considered in the present studies, might potentially play a role in the described process (e.g., self-acceptance as conceptualized in the construct of self-directedness, [43–44]). The concept of self-acceptance, in fact, is closely related to self-forgiveness as it implies the ability to let go of inner criticism and struggles [43–44]. Therefore, it might be possible that high locomotion concerns may help self-acceptance of past wrongs by enhancing, through the movement toward the future, the ease to let go of inner criticism which in turn increases self-forgiveness. This possibility may deserve to be investigated by future studies.

The findings of the present research also support recent dual-process models of self-forgiveness [45–46], which assert that self-forgiveness entails both (1) making a decision to accept responsibility for wrongs, and (2) replace of self-condemning emotions with self-affirming emotions. In fact, locomotion was positively associated with self-forgiveness, being perhaps positively associated with both components of self-forgiveness. On the other hand, assessment was negatively associated with self-forgiveness, being perhaps negatively associated with emotional restoration of personal esteem.

This all sounds good for high locomotion, but we need to mention a potential fly in the ointment. It is possible that high locomotors’ urge for movement and change could lead them to take action for future goals irrespective of past wrongs and mistakes. Yes, they are more self-forgiving but, at the same time, they could be more susceptible to repeat errors and mistakes. In this vein, research has suggested that self-forgiveness for ongoing, wrongful behavior (e.g., smoking; gambling) may function as an alleviating strategy that reduce negative feelings associated to the wrongs committed by the self, thereby decreasing any behavioral learning from these wrongs [13, 40, 47–49]. Thus, locomotion tendencies to move *forward* enhances self-forgiveness, but may also lead to an underestimation of past errors and, as a consequence, the repetition of wrongs. This possibility needs to be investigated in future research. Furthermore, considering that the two regulatory mode orientations (locomotion and assessment) are orthogonal dimensions and may work together [26] as part of the same goal pursuit (self-forgiveness in this case), we might further develop the idea expressed above. Specifically, even though self-forgiveness has taken place, and thus the impact of negative emotions of guilt and shame are considerably reduced, high assessment concerns may lead individuals to still consider past errors in order to not repeat them in the future. Therefore, it is possible that locomotion concerns help individuals to forgive themselves, reducing the negative impact of guilt and shame allowing for movement toward the future, while in a second moment the assessment concerns help to keep in mind the teaching from past wrongs in order to not repeat them in the future. The investigation of the above hypothesis may be interesting not only for its theoretical implications, but also because it may have notable and relevant applicability. For instance, knowledge on how the two regulatory mode orientations work together (in predicting self-forgiving and learning by mistakes) can be beneficial when working with youths. A problem with young people, in fact, is that they are typically highly impulsive, and display low levels of self-control [50], which lead them to an higher insorgence of risky and uncontrolled behaviors [51–52], and to lower self-forgiveness tendencies [53–54]. A concomitant combination of high locomotion and high assessment might allow highly impulsive young students to face their everyday duties at school in the best way, by both helping self-forgiveness for the occurrence of possible mistakes and learning from them, thereby reducing the reiteration of wrongful behaviors in the future. Also this possibility might be considered in future research.

Another potential area for future research would be to test the same hypotheses as the present research but for other kinds of forgiveness, such as forgiving others rather than self-forgiveness. In this regard, research has found that locomotion increases the motivation to reconcile and decreases the influence on reconciliation of the feelings of negativity from the conflict. In contrast, assessment decreases the motivation to reconcile and increases the influence of the negative feelings from the conflict [55]. These forgiveness-of-others findings are consistent with what we found for self-forgiveness. Given the results of above studies, however, there may be cases where the assessment orientation and self-forgiveness become positively related. For instance, because high-assessors tend to feel guilty for their past wrongs they may more likely attempt to make amends by offering an apology, thereby possibly receiving forgiveness from their victims and ultimately increasing self-forgiveness [56]. On the contrary, assessment orientation may even prompt perpetrators to punish themselves, perhaps if attempts to making amends are improbable both because (a) victims deny their forgiveness to offenders and because (b) perpetrators perceive victims’ positive reactions towards them improbable. These hypotheses should be considered as hints for future research.

## Supporting information

S1 File Dataset of Study1. (SAV)

S2 File Dataset of Study2. (SAV)

S3 File Dataset of Study3. (SAV)

S4 File Dataset of Study4. (SAV)
